# Dynamic Profile of CD4^+^ T-Cell-Associated Cytokines/Chemokines following Murine Myocardial Infarction/Reperfusion

**DOI:** 10.1155/2019/9483647

**Published:** 2019-03-18

**Authors:** Dongsheng Yuan, Jinjun Tie, Zhican Xu, Guanya Liu, Xinyu Ge, Zhulin Wang, Xumin Zhang, Shiyu Gong, Gang Liu, Qingshu Meng, Fang Lin, Zhongmin Liu, Huimin Fan, Xiaohui Zhou

**Affiliations:** ^1^Research Center for Translational Medicine, Shanghai East Hospital, Tongji University School of Medicine, Shanghai 200092, China; ^2^Shanghai Heart Failure Research Center, Shanghai 200120, China; ^3^Department of Cardiovascular and Thoracic Surgery, Shanghai East Hospital, Tongji University School of Medicine, Shanghai 200092, China; ^4^Department of Child Internal Medicine, Shanghai Children's Medical Center, Shanghai Jiaotong University, Shanghai 200127, China; ^5^Department of Cardiology, Shanghai East Hospital, Tongji University School of Medicine, Shanghai 200092, China; ^6^Department of Heart Failure, Shanghai East Hospital, Tongji University School of Medicine, Shanghai 200092, China

## Abstract

CD4^+^ T-cells play crucial roles in the injured heart. However, the way in which different CD4^+^ T subtypes function in the myocardial infarction/reperfusion (MI/R) heart is still poorly understood. We aimed to detect the dynamic profile of distinct CD4^+^ subpopulation-associated cytokines/chemokines by relying on a closed-chest acute murine MI/R model. The protein levels of 26 CD4^+^ T-cell-associated cytokines/chemokines were detected in the heart tissues and serum of mice at day 7 and day 14 post-MI/R or sham surgery. The mRNA levels of IL-4, IL-6, IL-13, IL-27, MIP-1*β*, MCP-3, and GRO-*α* were measured in blood mononuclear cells. The protein levels of IL-4, IL-6, IL-13, IL-27, MIP-1*β*, MCP-3, and GRO-*α* increased in both injured heart tissues and serum, while IFN-*γ*, IL-12P70, IL-2, IL-1*β*, IL-18, TNF-*α*, IL-5, IL-9, IL-17A, IL-23, IL-10, eotaxin, MIP-1*α*, RANTES, MCP-1, and MIP-2 increased only in MI/R heart tissues in the day 7 and day 14 groups compared to the sham group. In serum, the IFN-*γ*, IL-23, and IL-10 levels were downregulated in the MI/R model at both day 7 and day 14 compared to the sham. Compared with the protein expressions in injured heart tissues at day 7, IFN-*γ*, IL-12P70, IL-2, IL-18, TNF-*α*, IL-6, IL-4, IL-5, IL-9, IL-17A, IL-23, IL-27, IL-10, eotaxin, IP-10, RANTES, MCP-1, MCP-3, and GRO-*α* were reduced, while IL-1*β* and MIP-2 were elevated at day 14. IL-13 and MIP-1*β* showed higher levels in the MI/R serum at day 14 than at day 7. mRNA levels of IL-4, IL-6, IL-13, and IL-27 were increased in the day 7 group compared to the sham, while MIP-1*β*, MCP-3, and GRO-*α* mRNA levels showed no significant difference between the MI/R and sham groups in blood mononuclear cells. Multiple CD4^+^ T-cell-associated cytokines/chemokines were upregulated in the MI/R hearts at the chronic stage. These results provided important evidence necessary for developing future immunomodulatory therapies after MI/R.

## 1. Introduction

Ischemic heart diseases remained the leading cause of death worldwide in the last decade [1]. The reopening of the occluded coronary artery by percutaneous coronary intervention (PCI) achieves blood reperfusion of the ischemic myocardium, while it impairs the previously ischemic tissue, which is a concept known as ischemia/reperfusion injury [2].

Cardiac injury post myocardial infarction/reperfusion (MI/R) triggers subsequent left ventricular (LV) remodeling and eventual severe heart failure (HF) or disability [3]. Multiple mechanisms, including progressive apoptosis of myocardial cells, neurohumoral activation, and oxidative stress, contribute to the activation of innate immune responses, initiate a cascade inflammatory reaction, and lead to deterioration, which results in scar formation and ventricle dilation remodeling [4, 5].

CD4^+^ T-cells, known as T helper cells (Th cells), play pivotal roles in chronic inflammation, and they are closely involved in the progression of various cardiovascular diseases [6]. Upon stimulation and activation, naïve CD4^+^ cells differentiate into distinct CD4^+^ subtypes, including Th1, Th2, Th9, Th17, Th22, and Tregs [7, 8], all with different functions, by producing multiple cytokines or chemokines [9]. Naïve CD4^+^ T-cells, stimulated by IL-12 and IFN-*γ*, can differentiate into Th1 cells, while IL-4 leads to the polarization of Th2 cells [10, 11]. Th1 cells can express IFN-*γ*, TNF-*α*, IL-2, IL-3, IL-6, GM-CSF, and T-bet (T-box21) and are mainly involved in cell-mediated immunity [10], whereas Th2 cells mainly express IL-4, IL-5, IL-13, IL-10, and GATA3 (GATA-binding protein 3) and are responsible for humoral-mediated immune responses [7]. The following reports identified new CD4^+^ subpopulations, including Th9, Th17, and Th22 cells, which mainly express IL-9, IL-17, and IL-22, respectively [12, 13]. CD4^+^ regulatory T cells (Treg cells), serving as a brake, can block excessive inflammation and tissue destruction [14, 15]. These T helper subsets orchestrate to tune immune homeostasis, and the disturbance of the balance of these cells leads to inflammatory disease and tissue destruction.

Evidence has demonstrated the protective or pathogenic effects of CD4^+^ T-cells in the ischemic myocardium [16]. RAG1-knockout (KO) mice or mice depleted of CD4^+^ T-cells showed decreased infarct size compared to the control mice after MI/R [17]. Accordingly, the adoptive transfer of CD4^+^ T-cells into RAG1-KO mice reversed the protective effect [17]. Moreover, mice without CD4^+^ T-cells retained a higher ejection fraction and microvascular reperfusion than wild-type mice in acute myocardial infarction/reperfusion (AMI/R) models [18]. On the other hand, activated CD4^+^ T-cells are indispensable for suitable collagen scar deposition, which avoids left ventricular rupture and dilation after myocardial infarction (MI) [19]. These studies confirmed the importance of T cells in cardiac remodeling. The following study showed that CD4^+^ cells infiltrated in the heart at day 1 post-MI, peaked at day 7, and maintained a high level till day 14 [20]. However, how different CD4^+^ T subtypes function in the MI/R heart is still poorly understood. Previous studies often focused on a subset of CD4^+^ T-cells or a single chemokine/cytokine in the MI/R heart [21], which is insufficient to show the comprehensive and consecutive function of distinct CD4^+^ T-cells. In addition, to develop optimal therapies to block the chronic inflammation post-MI/R, it is mandatory to understand the dynamic profile of accumulation of CD4^+^ T-cell-associated cytokines and chemokines following MI/R. In this study, we applied a closed-chest MI/R mice model to evaluate the chronic dynamic profile of CD4^+^ T-cell-associated cytokines and chemokines.

## 2. Materials and Methods

### 2.1. Mice

Eight-week-old male C57BL/6 mice (Shanghai SLAC Laboratory Animal Co. Ltd.) that weighed 18-20 g were raised under specific pathogen-free conditions in an environment with constant temperature (23-24°C), humidity (55 ± 5%), and light (1 : 1 of day : night). We applied the Animal Protection Stipulation (Regulations of Experimental Animal Administration that were issued by the State Committee of Science and Technology of China), and the experiments and procedures performed by us were approved by the Committee on the Ethics of Animal Experiments of Tongji University.

### 2.2. Establishment of Murine MI/R Model

**Figure 1:**
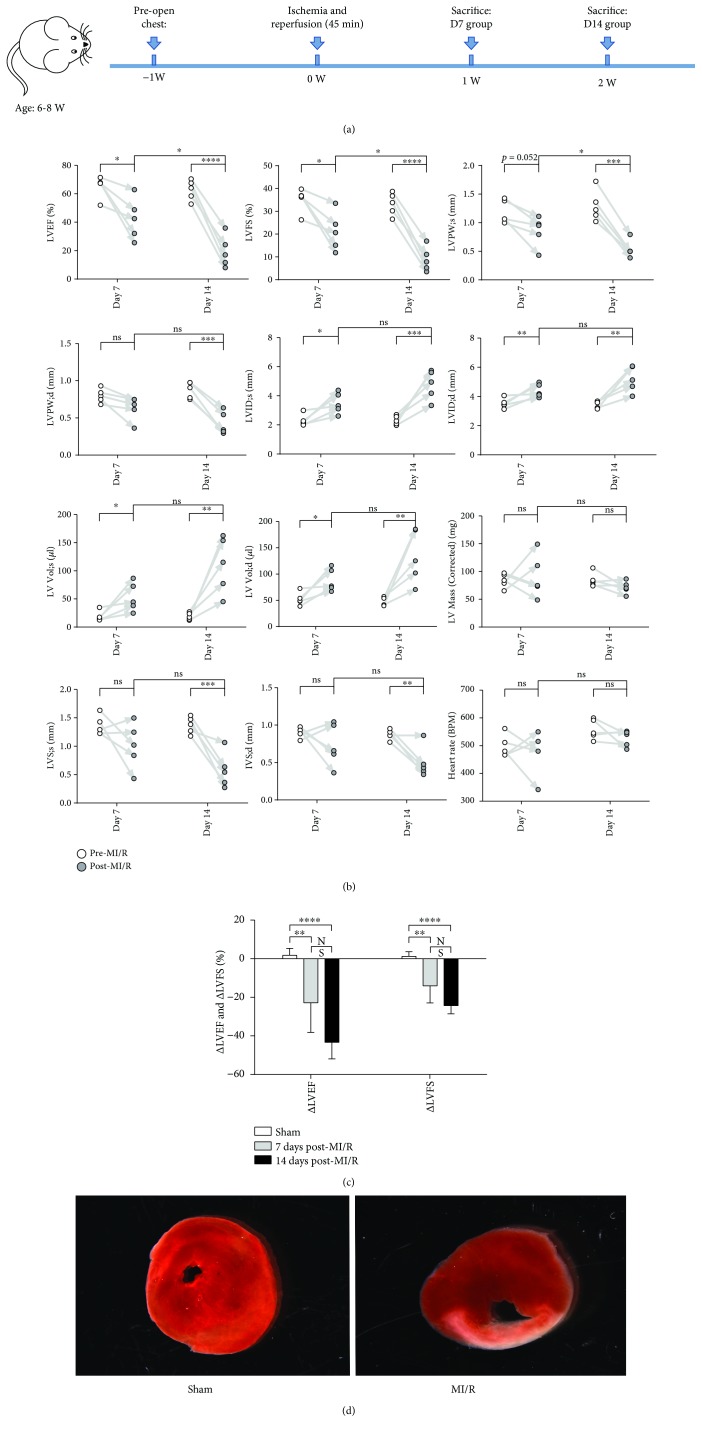
Cardiac dysfunction in mice post-MI/R. (a) The main time shaft of animal experiments was presented. (b) LVFS (%), LVPW (mm), LVID (mm), LV Vol (*μ*l), LV Mass (corrected) (mg), IVS (mm), and heart rates (BPM) were measured under transthoracic echocardiography pre-MI/R and post-MI/R at day 7 and day 14, respectively. ^∗^*P* < 0.05, ^∗∗^*P* < 0.01, ^∗∗∗^*P* < 0.001, and ^∗∗∗∗^*P* < 0.0001. (c) ΔLVEF (difference mean value between pre-MI/R and post-MI/R) and ΔLVFS (difference mean value between pre-MI/R and post-MI/R) were measured among the three groups. ^∗∗^*P* < 0.01 and ^∗∗∗∗^*P* < 0.0001. (d) The heart tissues were stained by TTC.

Mice were randomly divided into three groups: (1) sham group (*n* = 8); (2) MI/R day 7 group (*n* = 11); and (3) MI/R day 14 group (*n* = 12). A closed-chest mouse model of MI/R was used to avoid the intricacy effects of surgical trauma and nonspecific inflammation [22]. The protocol is presented in Figure 1(a). Shortly, after anesthesia (1.5% pentobarbital in N.S; intraperitoneal injection of 60 *μ*g/g body weight) and endotracheal intubation, the chest cavity was opened carefully, and the left anterior descending (LAD) coronary artery in the superficial portion of the left ventricle was located. An 8-0 silk suture with a U-shaped tapered needle was applied to pass under the LAD coronary artery, and the two ends of the suture were passed through a 0.5 mm PE-10 tubing. Next, the cavity was closed using a 5-0 silk suture, and the two ends of the 8-0 suture were exteriorized through each side of the chest wall and tucked under the skin. After 45 min, the 8-0 suture was cut near the chest wall to induce reperfusion. Identical operations without occlusion were executed in the sham group.

### 2.3. Determination of Cardiac Function

On day 7 and day 14 after AMI/R, transthoracic echocardiography was applied under isoflurane inhalation using the Vevo 2100 high-frequency high-resolution ultrasound system (Fujifilm VisualSonics Inc., Toronto, Canada). As previously delineated, the following values were recorded: left ventricular ejection fraction (LVEF, %), left ventricular fractional shortening (LVFS, %), left ventricular posterior wall thickness (LVPW, mm), left ventricular internal diameter (LVID, mm), left ventricular volume (LV Vol, *μ*l), left ventricular mass (LV Mass (corrected), mg), interventricular septum thickness (IVS, mm), and heart rates (BPM) [23]. ∆LVEF and ∆LVFS were calculated by subtracting pre-MI/R measurements from day 7/14 measurements after MI/R [24].

### 2.4. Tissue Histopathology

Heart tissues were harvested, fixed, embedded, and sectioned into 5 *μ*m slices for hematoxylin and eosin (H&E), Masson, and immunohistochemistry (IHC) staining on day 7 and day 14 after surgery. The IHC assay was performed as previously described [25]. Briefly, the paraffin-embedded slides were deparaffinized, endogenous peroxidase was blocked using 3% H_2_O_2_ diluted in ddH_2_O for 25 min, and the samples were blocked with 3% BSA for 30 min and incubated with anti-CD3 (ab16669, 1 : 100, Abcam), anti-CD4 (ab183685, 1 : 100, Abcam), anti-CD8 (#98941, 1 : 200, CST), anti-F4/80 (SC377009, 1 : 200, Santa Cruz Biotechnology), and anti-LY6G (MAB1037, 1 : 200, R&D) at 4°C for at least 12 hours. An HRP-labeled goat anti-rabbit IgG second antibody (074-1506, 1 : 200, KPL) was applied for 50 min at room temperature. For color development, the DAB kit (K5007, DAKO) was used, and the nucleus was counterstained with hematoxylin (Harris). An Upright Fluorescent Microscope (NIKON ECLIPSE CI-S, NIKON) and the NIKON DS-U3 imaging system were used to acquire images.

### 2.5. Myocardial Infarct Size

2,3,5-Triphenyl tetrazolium chloride (TTC) was applied to reveal the infarct size [26]. The heart was placed in a mouse heart matrix, kept at -80°C for 10 min, and then cut immediately in the matrix using a surgical blade along grooves. The slides were incubated in 1% TTC solution for 30 min at 37°C and incubated in 4% paraformaldehyde (PFA) for 30 min. Pictures were acquired using a stereological microscope (Leica M205FA).

### 2.6. Cytokine and Chemokine Profile Measurement

ProcartaPlex assay kit (Invitrogen, Cat. no. EPX260-26088-901) was used to measure the cytokine/chemokine profile. The mice serum and heart tissues were prepared and evaluated according to manufacturer's instructions. In brief, blood samples were collected in serum tubes, allowed to clot for 20–30 min at 20–25°C, and centrifuged at 1,000 × g for 10 min at 20–25°C. Heart tissues were smashed into the homogenate before centrifugation. A BCA kit (Pierce® BCA Protein Assay Kit, SE247581, product number: 23227 Thermo Fisher Scientific, USA) was used to quantify the protein levels of each sample. In this procedure, 25 *μ*l of samples and 25 *μ*l of Universal Assay Buffer were added into the bead-prepared wells. Next, the samples were incubated with shaking at room temperature for 60-120 min, and the beads were washed three times. Next, 25 *μ*l of Detection Antibody Mix, 50 *μ*l of Streptavidin-PE, and 120 *μ*l of reading buffer were added, followed by incubation and washing each time, as indicated by the protocol. Data were acquired on the Luminex™ 100/200 system.

### 2.7. Quantitative Real-Time Polymerase Chain Reaction (qRT-PCR)

qRT-PCR was applied for mRNA expression analysis [27]. PrimeScript RT Reagent Kits (TaKaRa, Japan) were used to reverse mRNA to cDNA. Fast Real-Time PCR System (7900HT; Applied Biosystems, Singapore) and SYBR Green MasterMix (Applied Biosystems, UK) were applied for qRT-PCR detection. The primers that we used to detect the expression change are as follows: mIL-4: 5′-GGTCTCAACCCCCAGCTAGT-3′, 5′-GCCGATGATCTCTCTCAAGTGAT-3′; mIL-6: 5′-TAGTCCTTCCTACCCCAATTTCC-3′, 5′-TTGGTCCTTAGCCACTCCTTC-3′; mIL-13: 5′-CCTGGCTCTTGCTTGCCTT-3′, 5′-GGTCTTGTGTGATGTTGCTCA-3′; mIL-27: 5′-CTGTTGCTGCTACCCTTGCTT-3′, 5′-CACTCCTGGCAATCGAGATTC-3′; mMIP-1*β*: 5′-TTCCTGCTGTTTCTCTTACACCT-3′, 5′-CTGTCTGCCTCTTTTGGTCAG-3′; mMCP-3: 5′-GCTGCTTTCAGCATCCAAGTG-3′, 5′-CCAGGGACACCGACTACTG-3′; mGRO-*α*: 5′-CTGGGATTCACCTCAAGAACATC-3′, 5′-CAGGGTCAAGGCAAGCCTC-3′; and m GAPDH: 5′-AGGTCGGTGTGAACGGATTTG-3′, 5′-GGGGTCGTTGATGGCAACA-3′.

### 2.8. Data Analysis

Data were analyzed using the SPSS software, version 11.0 (SPSS Inc., Chicago, USA, version 21.0 for Windows). One-way analysis of variance and nonparametric tests were used to assess potential statistically significant differences among groups. The results were displayed as the mean ± SD. The statistically significant level of *P* value was less than 0.05.

## 3. Result

### 3.1. Cardiac Dysfunction and Fibrosis after MI/R

Transthoracic echocardiography verified the cardiac dysfunction at days 7 and 14 post-MI/R. Both LVEF and LVFS in the MI/R model in the day 7 and day 14 groups were lower than before (Figure 1(b)). LVEF and LVFS in the day 14 group decreased greatly compared to that on day 7 post-MI/R. LVPW and IVS decreased at day 14 compared to before. LVID and LV Vol were higher at day 7 and day 14 post-MI/R. Furthermore, we measured ΔEF and ΔFS of the three groups. As shown in Figure 1(c), both ΔEF and ΔFS of MI/R in the day 7 and day 14 groups were lower compared to those in the sham group.

**Figure 2:**
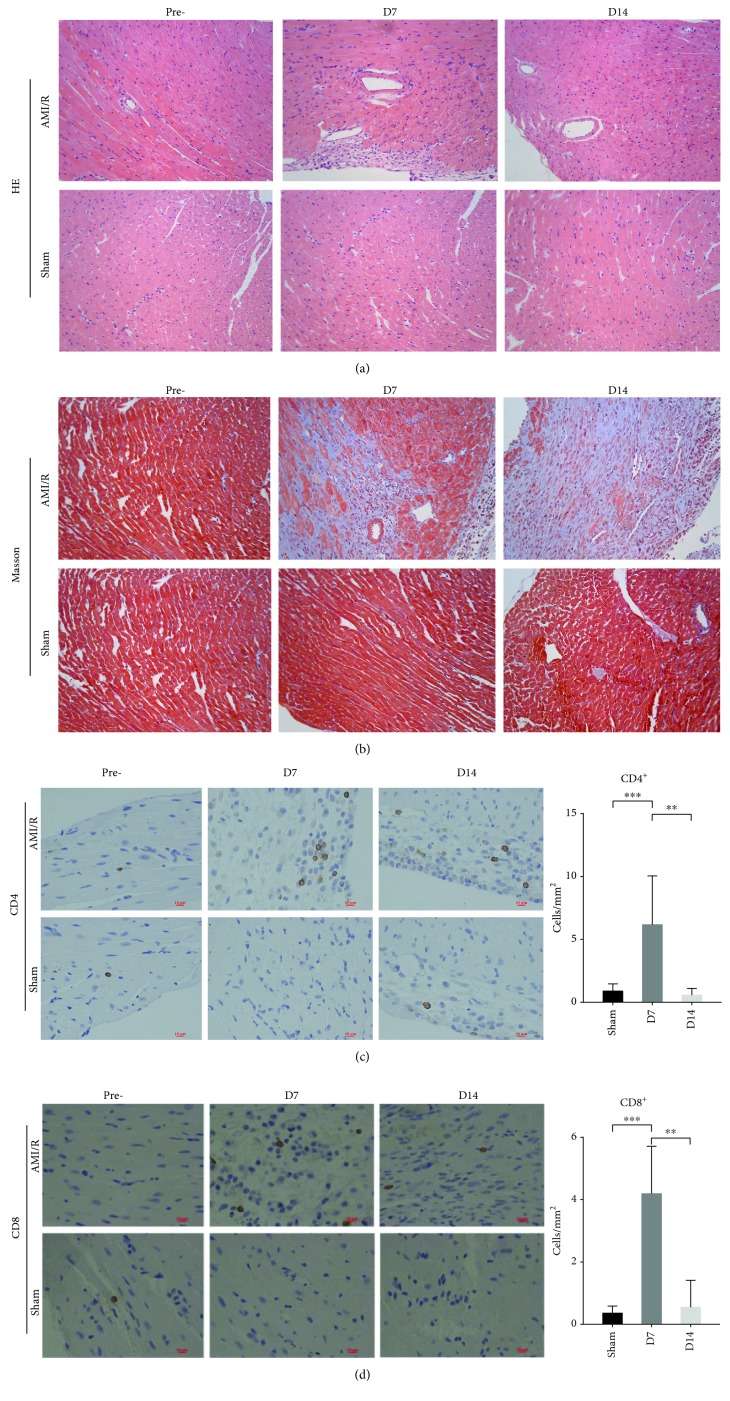
The morphology of heart tissues and CD4^+^/CD8^+^ T-cell infiltration post-MI/R. (a) Heart tissues were stained by H&E in magnifications 200x. (b) Heart tissues were stained by Masson in magnifications 200x. (c) CD4^+^ T-cells were detected by IHC in magnifications 400x. ^∗∗^*P* < 0.01 and ^∗∗∗^*P* < 0.001. (d) CD8^+^ T-cells were detected by IH in magnifications 400x. ^∗∗^*P* < 0.01 and ^∗∗∗^*P* < 0.001.

The infarct area of the heart sections in the MI/R groups was verified by TTC at day 3 after AMI/R (Figure 1(d)). Irregular arrangement of myocardial fibers and inflammatory cell infiltrations were identified via H&E staining (Figure 2(a)) and fibrosis via Masson staining results (Figure 2(b)) in the heart at day 7 and day 14. Further, IHC staining (Figures 2(c) and 2(d)) showed that CD4^+^ and CD8^+^ T-cells were increased in the heart tissues at day 7 compared to the sham group and decreased at day 14 compared to day 7.

### 3.2. Th1/Th2 Cell Cytokine Profile in Murine Hearts and Serum Post-MI/R

**Figure 3:**
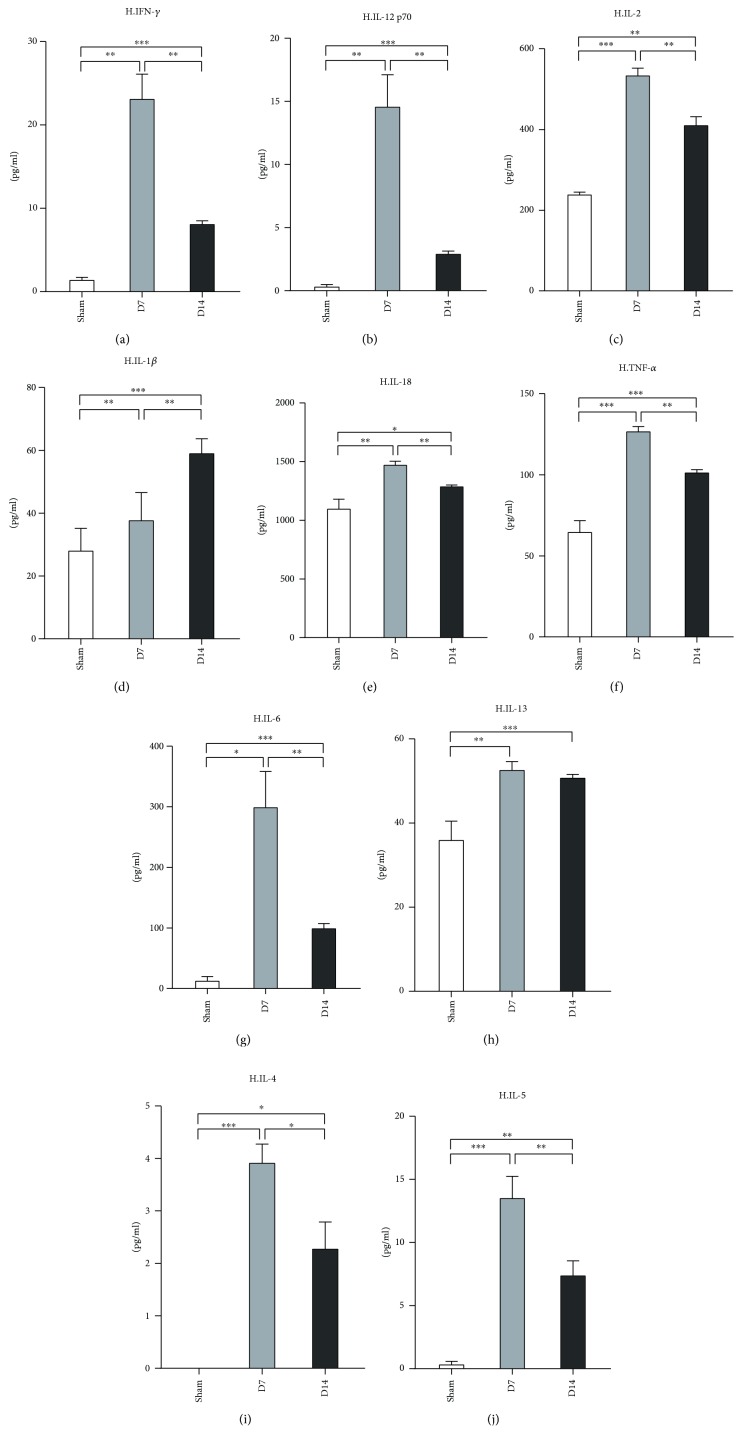
Measurement of Th1/Th2 associate cytokines in heart tissues at day 7 and day 14 post-MI/R. Sham group (*n* = 8), day 7 group (*n* = 11), and day 14 group (*n* = 12). The levels of IFN-*γ*, IL-12P70, IL-2, IL-1*β*, IL-18, TNF-*α*, IL-6, IL-23, IL-4, and IL-5 were measured. ^∗^*P* < 0.05, ^∗∗^*P* < 0.01, and ^∗∗∗^*P* < 0.001.

Next, we evaluated Th1/Th2-associated cytokine profile in the mouse heart tissues post-MI/R. The protein levels of IFN-*γ*, IL-12p70, IL-2, IL-18, IL-6, TNF-*α*, IL-6, IL-4, and IL-5 increased at day 7 post-MI/R compared to the sham group (Figure 3). In contrast, those cytokine levels were decreased at day 14 compared to day 7. The protein expression of IL-1*β* increased gradually from day 7 to day 14, and the GM-CSF level was upregulated in the heart at day 7 compared to the sham group (Figure 3(d)). The IL-13 level was upregulated at day 7 and maintained a high level until day 14 (Figure 3(h)).

**Figure 4:**
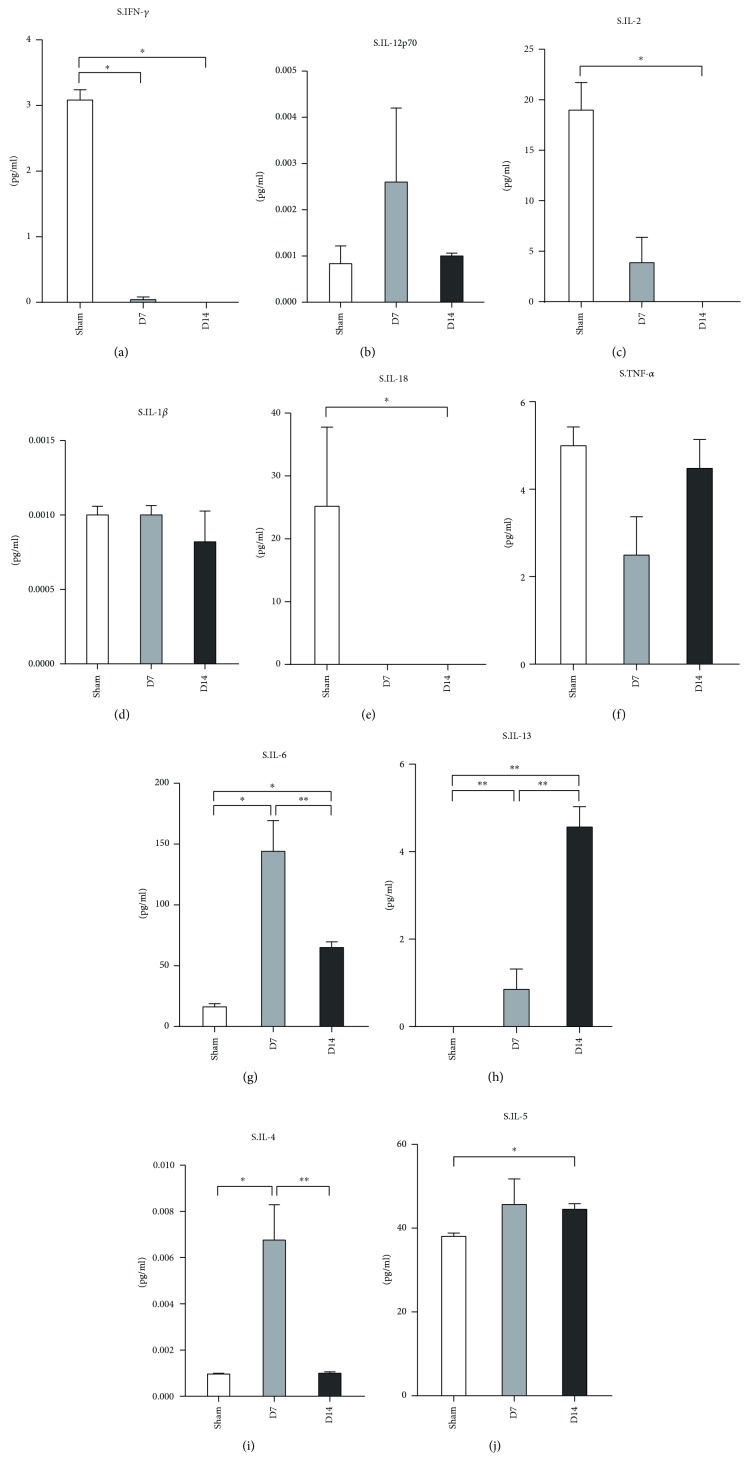
Measurement of Th1/Th2 associate cytokines in serum at day 7 and day 14 post-MI/R. Protein levels of IFN-*γ*, IL-12P70, IL-2, IL-1*β*, IL-18, TNF-*α*, IL-6, IL-23, IL-4, and IL-5 were measured in serum from the sham group (*n* = 8), the day 7 group (*n* = 11), and the day 14 group (*n* = 12). ^∗^*P* < 0.05 and ^∗∗^*P* < 0.01.

We further determined the expression of Th1/Th2-related cytokines in the serum. Protein levels of Th1-associated cytokines including IFN-*γ* and IL-2 were lower than those in the sham group at day 7 and day 14 (Figures 4(a) and 4(c)). The levels of IL-6, IL-13, and IL-4 were increased at day 7 (Figures 4(g)–4(i)). At day 14, the level of IL-13 increased compared to day 7, while the levels of IL-6 and IL-4 reduced. There were no differences in the IL-12p70, IL-1*β*, and TNF-*α* expression levels among the groups (Figures 4(b), 4(d), and 4(f)).

### 3.3. Cytokines Associated with Th9/Th17/Th22 and Treg Cells in Murine Model following MI/R

**Figure 5:**
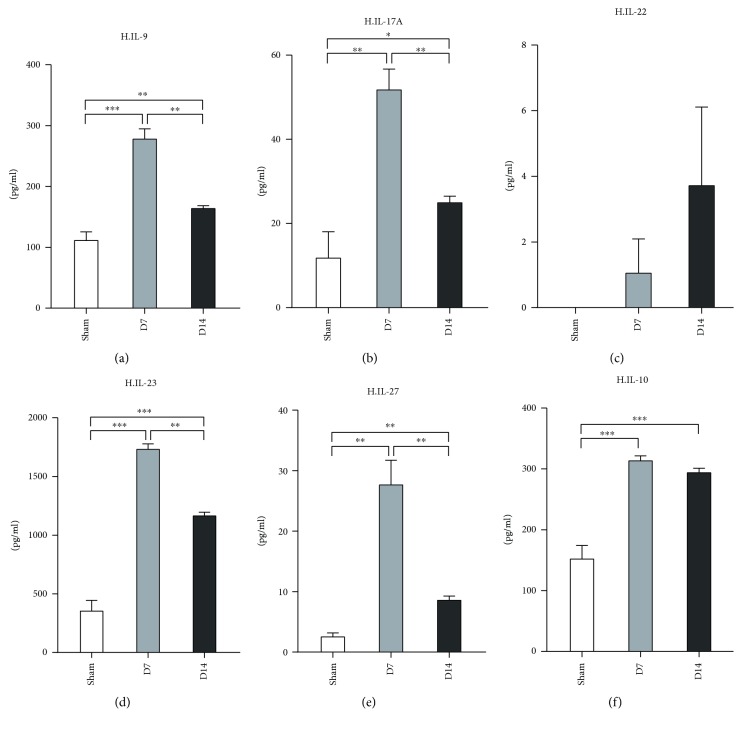
Measurement of Th9, Th17, Th22, and Treg associate cytokines in heart tissues at day 7 and day 14 post-MI/R. Sham group (*n* = 8), day 7 group (*n* = 11), and day 14 group (*n* = 12). ^∗^*P* < 0.05, ^∗∗^*P* < 0.01, and ^∗∗∗^*P* < 0.001.

Next, we detected the cytokine expression profile of Th9/Th17/Th22 and Treg cells in murine hearts post-MI/R. Our data revealed that the protein levels of IL-9, IL-17A, IL-23, and IL-27 showed the same expression pattern in which all four were higher at both day 7 and day 14 than the sham group but dramatically decreased at day 14 when compared to day 7 (Figures 5(a), 5(b), 5(d), and 5(e)). In comparison with the sham, the IL-10 level increased at day 7 and day 14.

**Figure 6:**
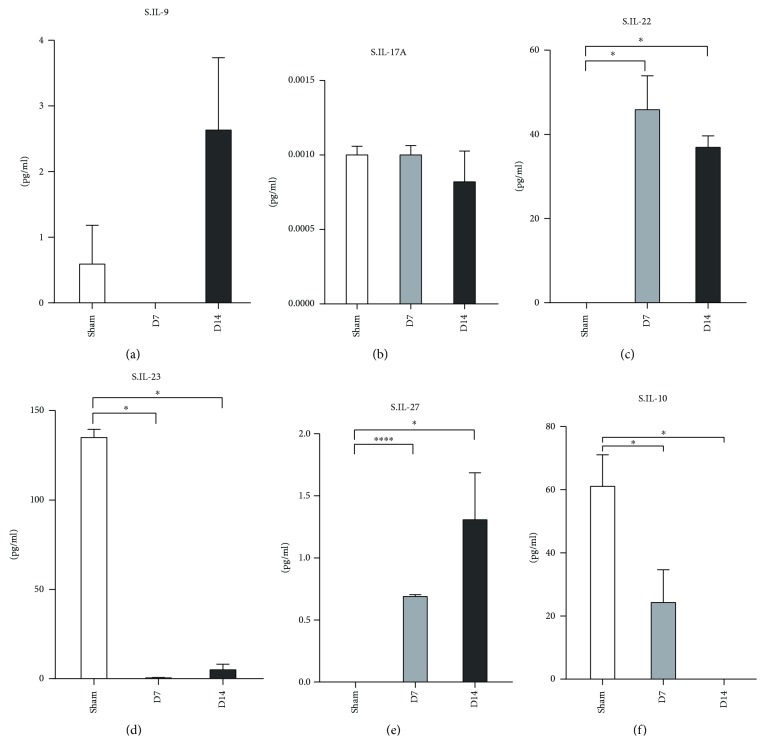
Measurement of Th9, Th17, Th22, and Treg associate cytokines in serum at day 7 and day 14 post-MI/R. Sham group (*n* = 8), day 7 group (*n* = 11), and day 14 group (*n* = 12). ^∗^*P* < 0.05 and ^∗∗∗^*P* < 0.001.

Furthermore, the serum levels of IL-10 and IL-23 decreased at day 7 and day 14 compared to the sham group (Figures 6(d) and 6(f)). The levels of IL-22 and IL-27 increased at day 7 and day 14 post-MI/R (Figures 6(c) and 6(e)).

### 3.4. Chemokine Expression Profile in Heart Tissue and Serum following Infarction and Reperfusion

**Figure 7:**
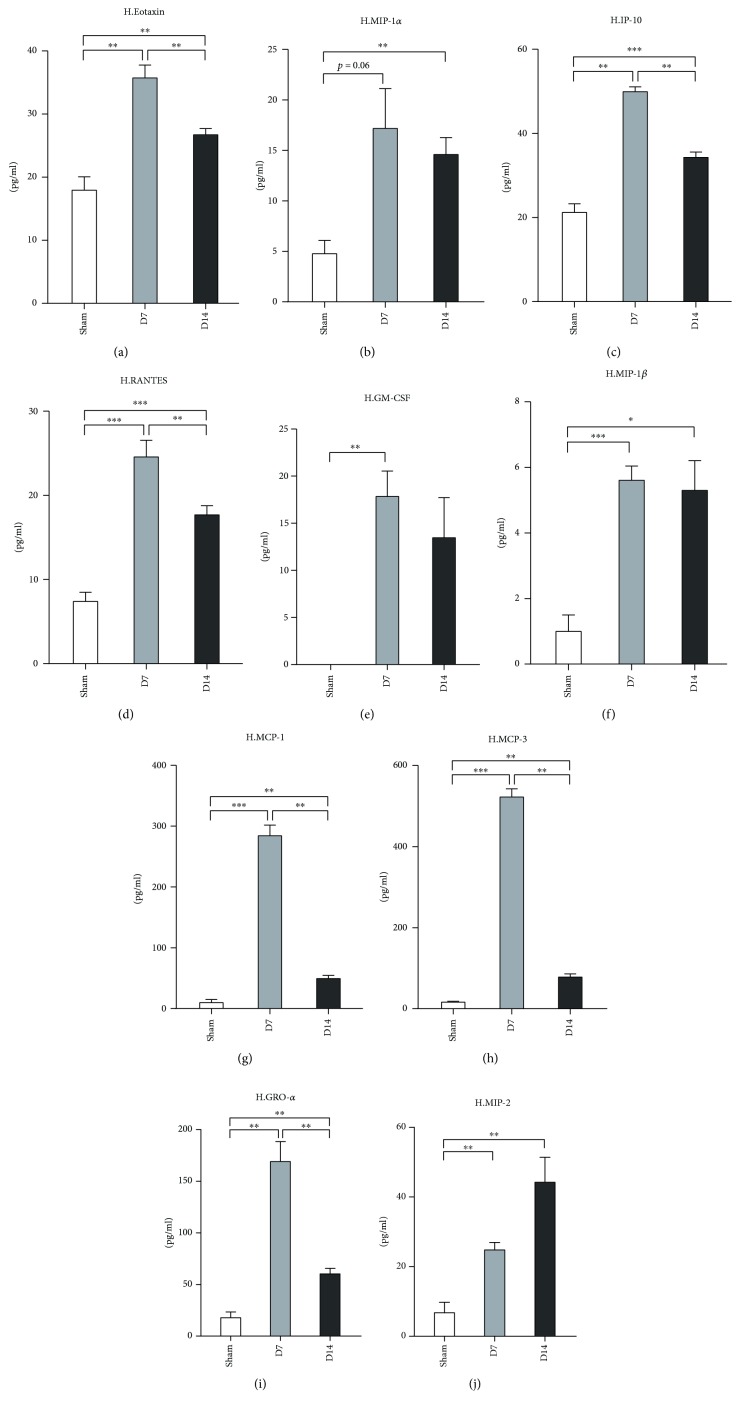
Chemokine profile in heart tissue at day 7 and day 14 post-MI/R. Sham group (*n* = 8), day 7 group (*n* = 11), and day 14 group (*n* = 12). ^∗^*P* < 0.05, ^∗∗^*P* < 0.01, and ^∗∗∗^*P* < 0.001.

The migration of CD4^+^ T-cells into the injured area is thought to depend on the chemokine production [28]. We found that the protein levels of eotaxin (CCL11), IP-10 (CXCL10), RANTES (CCL5), MCP-1 (CCL2), MCP-3 (CCL7), and GRO-*α* (CXCL1) were upregulated at day 7 and downregulated at day 14 (Figures 7(a), 7(c), 7(d), 7(g), 7(h), and 7(i)) in the injured heart tissue. The MIP-1*β* level at day 7 was higher than that in the sham group and was maintained until day 14 (Figure 7(f)). Only the level of MIP-2 increased constantly after MI/R from day 7 to day 14 (Figure 7(j)). The level of MIP-1*α* was only increased at day 14 (Figure 7(b)).

**Figure 8:**
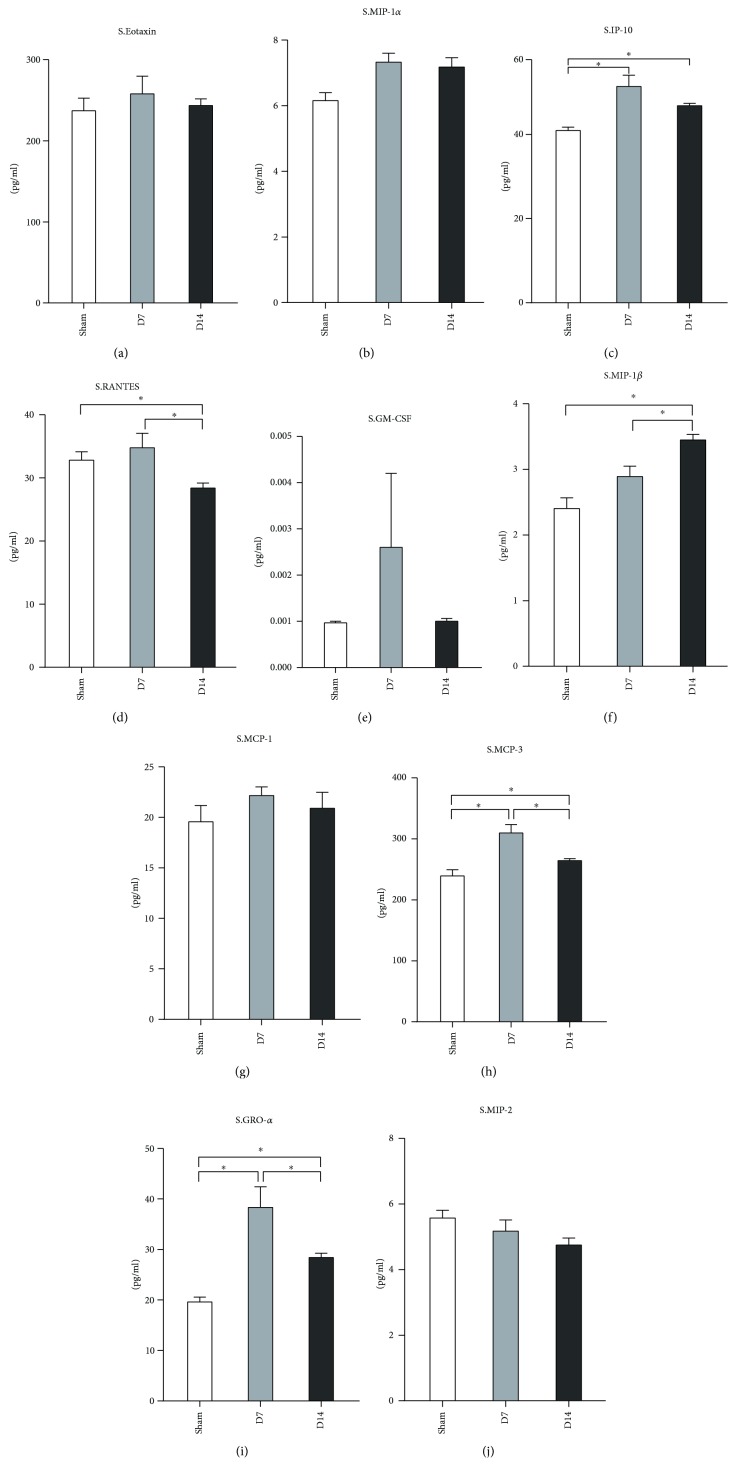
Chemokine profile in serum at day 7 and day 14 post-MI/R. Sham group (*n* = 8), day 7 group (*n* = 11), and day 14 group (*n* = 12). ^∗^*P* < 0.05.

We also detected the chemokine expression profile in the serum after MI/R. As presented in Figure 8, both protein levels of GRO-*α* and MCP-3 in the serum were upregulated at day 7 and day 14, which is similar to their expression patterns in the heart tissues. The level of MIP-1*β* was increased at day 14, while the RANTES level decreased at day 14 (Figures 8(d) and 8(f)) compared to the sham group and the day 7 group. The expression of IP-10 at day 7 and day 14 was higher than that in the sham group but declined at day 14 compared to day 7 (Figure 8(c)). Eotaxin, MIP-1*α*, and MCP-1 showed no difference among the three groups (Figures 8(a), 8(b), and 8(g)).

### 3.5. The mRNA Levels of IL-4, IL-6, IL-13, IL-27, MIP-1*β*, MCP-3, and GRO-*α* in Mononuclear Cells in Blood

**Figure 9:**
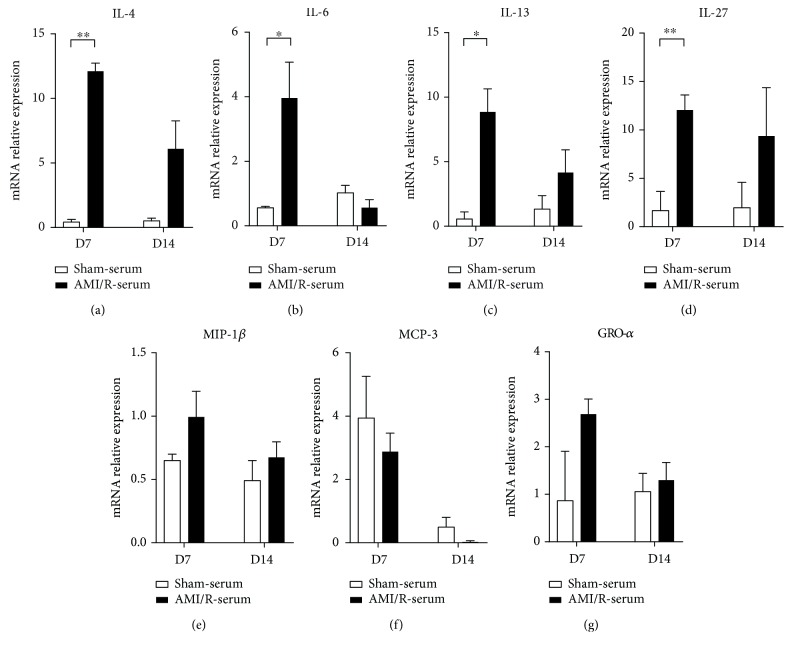
The mRNA levels of IL-4, IL-6, IL-13, IL-27, MIP-1*β*, MCP-3, and GRO-*α* in mononuclear cells in blood. Sham group (*n* = 8), day 7 group (*n* = 11), and day 14 group (*n* = 12). ^∗^*P* < 0.05 and ^∗∗^*P* < 0.01.

To further reveal whether the increase of IL-4, IL-6, IL-13, IL-27, MIP-1*β*, MCP-3, and GRO-*α* protein levels in the serum was attributed to their release from the injured heart or production by blood mononuclear cells, we tested their mRNA expressions in blood mononuclear cells from the three groups. The mRNA levels of IL-4, IL-6, IL-13, and IL-27 were increased in the day 7 and day 14 groups compared with the control group (Figures 9(a)–9(d)). However, MIP-1*β*, MCP-3, and GRO-*α* mRNA levels showed no significant difference between the MI/R and control groups (Figures 9(e)–9(g)).

## 4. Discussion

The present study provides a comprehensive analysis of the temporal dynamics of CD4^+^ T-cell-related cytokines/chemokines in injured heart tissues and serum in the murine AMI/R model. In this study, we applied a closed-chest MI/R model instead of the traditional models (anesthetized open-chest method) [22]. Previously, Michael et al. [29] verified that the surgical trauma associated with the open-chest method resulted in significant background levels of inflammatory mediators within 7 days post operation. The open-chest method models simulate the clinical condition of cardiac MI/R during coronary artery bypass grafting (CABG) procedures, whereas the closed-chest model imitates the condition of cardiac MI/R during PCI [30]. Thus, we used the closed-chest MI/R model to simulate patients suffering from MI/R in clinical settings. Two methods were utilized to affirm the success of our MI/R model: TTC staining and transthoracic echocardiography. The gray infarct area in heart sections and decreased levels of LVEF, LVFS, or other index confirmed that the surgical methods described above were efficient.

Chronic inflammation mediated by T cells has been confirmed to be a major player in the etiology and development of ventricular remodeling post-MI/R [31]. Subsequent studies showed the role of CD4^+^ T-cells in the pathology and healing of the heart after MI or MI/R [16, 19, 32]. Consistent with the previous report that T cells were enriched 5- to 10-fold in the MI/R heart at day 7 compared to the healthy control [20], our IHC data showed that more CD4^+^ T-cells infiltrated in the MI/R hearts at day 7 and day 14 than the sham group. Compared to the day 7 group, the injured hearts at day 14 revealed a decreased number of CD4^+^ T-cells. These findings further confirmed the involvement of CD4^+^ T-cells in cardiac chronic inflammation post-MI/R.

CD4^+^ T-cells, with multiple subtypes, exert their functions by producing distinct effectors. A previous study showed that CD4^+^ T-cells in murine injured heart tissue are mainly Th1 polarized [20]. Both levels of interleukin IL-6 and tumor necrosis factor- (TNF-) *α* were elevated in MI patient serum compared to controls on day 1 and day 6 post-MI/R [33–35]. In murine MI models, IL-6 and TNF-*α* levels increased 1 week after MI and decreased rapidly thereafter in the infarct zone in mice models [29]. Consistent with this report, we found a similar expression of both IL-6 and TNF-*α* in the hearts of our MI/R model. We also showed that other Th1-associated cytokines including IFN-*γ*, IL-2, IL-12p70, IL-18, and IL-1*β* present a similar expression pattern to TNF-*α* and IL-6, while IL-1*β* increased continually till day 14. In addition, we determined the expression of these cytokines in serum. Only IL-6 was increased at 1 week and decreased at 2 weeks in the serum.

IFN-*γ* is a characteristic cytokine produced by Th1 cells. It is reported that IFN-*γ* expression decreased in the serum of patients within 24 hours after the onset of acute MI [36]. Furthermore, IFN-*γ* concentration decreased at day 3 in the peripheral blood of subjects suffering from AMI and under PCI treatment [37]. While no difference was found in the serum IFN-*γ* levels between patients under the chronic recovery period post-MI and the control groups [38], in the murine models, the IFN-*γ* level increased in the cardiac tissue from one week to one month after MI [39]. Our results from the MI/R model demonstrated the high IFN-*γ* expression in heart tissues till day 14. In addition, the IFN-*γ* level at day 14 was lower than that at 7 days in MI/R cardiac tissues. Therefore, reperfusion may help to attenuate the IFN-*γ*-mediated inflammation in murine models compared to nonreperfusion.

IL-18, which belongs to the IL-1 cytokine super family, was previously reported to play an important role in immune, infectious, and inflammatory diseases due to its capacity to activate established Th1 cells to produce IFN-*γ* in the presence of IL-12 [40, 41]. Multiple cell types in the heart, including cardiomyocytes, can produce IL-18 in response to injury [42, 43]. High levels of IL-18 in the serum increased cardiovascular disease mortality, such as heart failure and AMI/R [44]. Patients with AMI exhibit a significant increase of IL-18 within 48 hours in the serum [45]. In murine MI models, IL-18 production was elevated in heart tissues and the serum at day 7 post operation [43]. Distinct from the previous data, we found that IL-18 was upregulated in heart tissues at both day 7 and day 14 but not in the serum compared to the sham group, which is similar to the expression pattern of IFN-*γ*. Therefore, IL-18 and IFN-*γ* may exert their roles synergistically in chronic inflammation and remodeling of the MI/R heart for a long time.

IL-2 was known as a crucial T cell growth and differentiation factor [46]. It was reported that the IL-2 level was lower at day 14 in serum samples of AMI patients than in the control [47], which suggests that it may act in the AMI acute phase. Since the life spans of mice and humans are different [48], we detected chronic inflammatory responses at day 7 and day 14 post murine MI/R. Our results showed that a high level of IL-2 was present in murine heart tissues but not in the serum after MI/R at day 7 and day 14; this indicated that IL-2 also contributes to the chronic pathogenesis of the injured heart after MI/R. Moreover, our results support the notion that the peripheral IL-2 level may not indicate the cardiac condition. High levels of IL-2 in the local tissue may exert its role by triggering the differentiation and proliferation of other T cells.

Multiple studies have demonstrated the importance of Th1/Th2 balance in diseases [49–51]. Th2 cells mainly produce IL-4, IL-5, and IL-13. In the peripheral blood, IL-4 concentrations increased in patients at day 3 after AMI and PCI treatment compared to the healthy control [37]. It is reported that a deficiency of IL-5 can result in accelerated atherosclerosis in ApoE^−/−^ mice [52]. A subsequent study showed that the IL-5 level was elevated in the peripheral blood of patients within 24 hours of acute MI [53]. A recent study has shown the protective role of IL-13 in cardiac wound healing within the infarct zone [54]. Our results revealed that the protein concentrations of IL-4, IL-5, and IL-13 all increased in MI/R hearts at day 7 and day 14 compared to the sham group. In contrast to the lower or equal levels of Th1-related cytokines in the serum, Th2-associated cytokines such as IL-5 and IL-13 were significantly upregulated in the serum at day 14 in the MI/R mice compared to the sham. These results indicated that Th2 cells may be involved in the healing of the injured heart. IL-13 was reported to modify wound healing within the infarct zone through regulating leukocyte recruitment and inducing M2-like monocyte/macrophage differentiation [54]. Further exploration is needed to determine how IL-5 is involved in this process.

IL-17, a proinflammatory cytokine that is mainly produced by CD4^+^ T-cells (Th17 cells), is known for its chemotactic and activating action on neutrophils in injured hearts [55]. A recent study has indicated that circulating Th22 and Th9-type responses may play a potential role in the onset of acute coronary syndromes [56]. Th22 and Th17 cells were significantly increased in patients who were subjected to AMI and unstable angina compared with those with stable angina and healthy controls [57]. In addition, the serum IL-17A level significantly increased in patients within 4 hours post-AMI/R compared to healthy controls [34], and IL-22 expression was upregulated in the peripheral blood within 12 hours in patients with AMI [56]. Another study revealed a significant increase in the number of peripheral Th22 cells and the protein levels of IL-22 and IL-9 in patients with acute coronary syndromes compared with those in the stable angina pectoris and control groups [56]. The plasma IL-22 level was significantly elevated in acute myocardial infarction (AMI) and unstable angina (UA) patients within 24 hours [57]. Moreover, the mean level of IL-27 in the serum of the AMI and UA groups was significantly higher than that of the control [58], which indicates that the higher serum level of IL-27 was associated with the incidence of ischemic heart disease (IHD). Our results showed that the protein levels of IL-9, IL-17, IL-23, and IL-27 were all increased greatly in the hearts at day 7 and day 14 post-MI/R. However, compared with the day 7 group, the expression of these four cytokines in the heart decreased significantly at day 14. Nevertheless, no differences in these cytokines except IL-23 were found in serum of the three groups. These results suggested that IL-9-, IL-17-, IL-23-, and IL-27-mediated inflammatory responses exist in the injured heart till a chronic phase at day 14 post murine MI/R.

Recent evidence has confirmed the contribution of Tregs to the healing of injured hearts [59]. Tregs exert their roles by secreting soluble cytokines, such as IL-10; the latter has been reported to exhibit an inhibitory effect on the inflammation and regulation of extracellular matrix metabolism [60, 61]. The upregulation of IL-10 reduces infarct size and improves cardiac function [62]. A previous report has reported that IL-10 increased at day 7 and day 30 in the serum of AMI patients [63]. We showed that the IL-10 levels increased in cardiac tissues, while they decreased in the peripheral serum at 1 week and 2 weeks post myocardial infarction, compared with that in the control group. The migration of Tregs and Th2 cells to the injured heart may explain the distinct profile of IL-10 expression in the serum and heart tissue. Long-term follow-up visits and detection may help to elucidate the relationship between these cytokine levels and prognosis.

Chemokines play an important role in cardiac repair and remodeling after myocardial infarction [64]. Chemokines attract a large number of immune cells and participate in the development of the innate and acquired immune responses [65]. Mice with heart failure and inducible cardiomyocyte-specific knockout of the sarcoplasmic reticulum Ca^2+^-ATPase (SERCA2KO) exhibited an increase in circulating levels of eotaxin, granulocyte-colony stimulating factor (G-CSF), monocyte chemoattractant protein-1 (MCP-1), and macrophage inflammatory protein-1beta (MIP-1*β*) [66]. A pilot study showed no difference in the serum levels of eotaxin, MCP-1, RANTES, and MIP-1*α* post-AMI within 24 hours in patients and the control [67]. The serum levels of GM-CSF, eotaxin, MCP-1, MCP-1*α*, MCP-1*β*, and RANTES increased at 2-3 weeks after AMI compared to the healthy control [68]. The serum GRO-*α* concentration increased in patients with congestive heart failure [69]. The MCP-3 level in the serum or heart tissue has not been studied till now in MI/R. Our data revealed that MI/R triggered high protein levels of eotaxin, MCP-1, MIP-1*α*, MIP-1*β*, RANTES, and MCP-3 at day 7 and at day 14 in heart tissues. Compared to day 7, their expressions decreased at day 14. These results suggested that those chemokines highly expressed in injured heart tissues may participate in the repairing and remodeling of the heart after AMI/R. It is noteworthy that both GRO-*α* and MCP-3 revealed a similar expression pattern in the serum and heart tissues, which indicates that we may determine GRO-*α*- and MCP-3-related responses in the heart indirectly via their serum levels.

In our murine MI/R model, most of the CD4^+^ T-cell-associated inflammatory factors are upregulated at day 7, and they maintain a high level till day 14 in the heart, while their expressions are relatively low in the serum. Therefore, it is difficult to predict the responses mediated by T cell effectors in the injured hearts by assessing their levels in the serum. Our qRT-PCR results further confirmed that MIP-1*β*, MCP-3, and GRO-*α* mRNA expression levels did not increase in blood mononuclear cells post-MI/R compared to the sham, while their protein levels were all upregulated in the heart, indicating that the increased MIP-1*β*, MCP-3, and GRO-*α* protein expressions in the serum may attribute to their release from the injured heart, and their serum levels may be used to predicate their expression in the injured heart at the chronic stage. Many studies have shown that T cells migrated to the injured heart and exerted their role locally [70]. Because of the difficulties in obtaining MI/R heart samples from patients, it is necessary to explore the association of the expression of multiple cytokines and chemokines between the serum and tissues. Further studies are needed to clarify this relationship in patients.

In conclusion, T-cell-associated cytokines/chemokines including IL-6, IL-1*β*, TNF-*α*, IL-4, IFN-*γ*, IL-5, IL-18, IL-2, IL-12p70, IL-13, IL-9, IL-17A, IL-23, IL-27, IL-10, IP-10, RANTES, MIP-1*β*, MCP-3, GRO-*α*, eotaxin, MIP-1*α*, MCP-1, MCP-3, and MIP-2 were all upregulated in the heart at day 7 and day 14 in the closed-chest murine MI/R model compared to the sham. The protein levels of IFN-*γ*, IL-2, IL-18, IL-10, and IL-23 decreased in the serum of the MI/R group compared with those in the sham serum. IL-5, IL-13, IL-6, and MIP-1*β* levels were elevated in the serum at day 14 post-MI/R. Moreover, the increased protein levels of MIP-1*β*, MCP-3, and GRO-*α* in the serum may indicate their accumulation in the chronic injured heart post-MI/R.

## Data Availability

The data used to support the findings of this study are available from the corresponding author upon request.
